# Joint Transcriptomic and Metabolomic Analysis Reveals Differential Flavonoid Biosynthesis in a High-Flavonoid Strawberry Mutant

**DOI:** 10.3389/fpls.2022.919619

**Published:** 2022-06-28

**Authors:** Yuanxiu Lin, Guoyan Hou, Yuyan Jiang, Xiaoyang Liu, Min Yang, Liangxin Wang, Yu Long, Mengyao Li, Yunting Zhang, Yan Wang, Qing Chen, Yong Zhang, Xiaorong Wang, Haoru Tang, Ya Luo

**Affiliations:** ^1^College of Horticulture, Sichuan Agricultural University, Chengdu, China; ^2^Institute of Pomology and Olericulture, Sichuan Agricultural University, Chengdu, China

**Keywords:** strawberry, proanthocyanidins, anthocyanins, metabolites, high-flavonoid content mutant

## Abstract

The enriched phenolic content attributes to the promising health benefit of strawberry fruits. On behalf of screening and seeking the breeding material with high phytochemical composition, a mutant (MT) of strawberry ‘Benihoppe’ (WT) with high total flavonoid content (TFC), especially anthocyanins and proanthocyanidins (PAs), was identified in this study. To investigate the possible reason for these disparities during strawberry fruit development, an integrated transcriptomic and metabolomic analysis was conducted using these two specific materials. As a result, a total of 113 flavonoid compounds were detected, a specific anthocyanin, namely, petunidin 3-*O*-rutinoside was detected for the first time in strawberry. By comparing with the WT fruits, a significant reduction of petunidin 3-*O*-rutinoside while around 24 times higher of cyanidin 3-*O*-rutinoside in MT fruits were observed. However, the cyanidin 3-glucoside content did not show obvious changes between MT and WT fruits, the pelargonidin and its derivatives were up-regulated only in partial red (PR) stage, but not in large green (LG) and fully red (FR) stages. Notably, the PAs such as procyanidin B2, procyanidin A1, catechin, gallocatechin gallate, epigallacatechin, and theaflavin were markedly up-regulated in MT. These results revealed a differential flavonoid biosynthesis between the two detected strawberry genotypes. A joint analysis with transcriptome data explained the up-regulation of cyanidin-based anthocyanins and PAs were caused by the down-regulation of *F3*′*5*′*H*, and up-regulation of *F3'H* and *LAR* expression, which might be regulated by the upregulation of potential TFs such as C3H, MADS, and AP2/ERF TFs. Metabolite correlation analysis suggested that it was PAs but not anthocyanins strongly correlated with the total phenolic content (TPC), indicated that PAs might contribute more to TPC than anthocyanins in our detected strawberry samples. This study not only potentially provided a new mutant for further breeding program to obtain high flavonoid content strawberry but also gave insights into strawberry flavonoid metabolic regulatory network, laid the foundation for identifying new flavonoid regulators in strawberry.

## Introduction

Strawberry (*Fragaria* × *ananassa* Duch.), being an important source of health promoting compounds, is widely produced and consumed in the fresh fruit market all over the world. It has been highly regarded as a functional food conferring multiple health benefits beyond basic nutrition, as supported by the increasing evidences on its antioxidant activity, anti-cancer, anti-inflammatory, or antihypertensive effects (Basu et al., 2014; Gasparrini et al., 2018). As strawberry fruits contain considerable quantities of phenolic compounds, which were shown to be the predominate contributor in antioxidant activities (Aaby et al., 2012).

The phenolic compounds belonging to secondary metabolites are not only involved in defense against ultraviolet radiation or aggression by pathogens but also contributing to the attractive color, flavor (bitterness), and oxidative stability of food (Pandey and Rizvi, 2009). The most abundant phenolic compounds found in fruits can be divided into several classes, such as flavonoids, phenolic acids, stilbenes, lignans, and tannins or PAs (Vasantha Rupasinghe et al., 2014). Particularly, the primary group of phenolic compounds in strawberry which historically has attracted most attention is the flavonoids, especially anthocyanins (Aaby et al., 2012). In addition to possess promising health benefits, the concentration and composition of anthocyanins are also responsible for the red color, which is one of the most important sensory qualities of strawberry fruits. Besides, strawberry fruits also contain high concentration of PAs, which play essential roles in fruit growth and development, resistance of plants against (a) biotic stresses, as well as the influence of astringency of fruits. More important, PAs are considered beneficial to human health as well (Buendía et al., 2010; Yu et al., 2020). More and more researches gain the attention to regulatory mechanism of Pas; however, the content of PAs decrease with growing maturity of the strawberry fruit regrettably (Zhang et al., 2018).

Both anthocyanins and PAs are the products of the general phenylpropanoid pathway derived flavonoid pathway, they share the common upstream steps of synthesis The basic skeleton of flavonoids is synthesized starting from malonyl-CoA and 4-coumaroyl-CoA. Subsequently, the naringenin (colorless flavanone) is produced by the catalysis of chalcone synthase (CHS) and chalcone isomerase (CHI). Thereafter, naringenin is either oxidized to form the isoflavones, or to produce dihydrokaempferol (colorless dihydroflavonol) under the catalyzation of flavanone 3-hydroxylas (F3H). Furtherly, the dihydrokaempferol is directed to the flavonols branch by the action of flavonol synthase (FLS), or converted to the leucoanthocyanidins catalyzed by the dihydroflavonol 4-reductase (DFR). The leucoanthocyanidins are furtherly oxidized to form anthocyanidins by the actions of anthocyanidin synthase (ANS) or leucoanthocyanidin dioxygenase (LDOX). Finally, the leucoanthocyanidins and anthocyanidins are subsequently catalyzed by leucoanthocyanidin reductase (LAR) and anthocyanidin reductase (ANR), leading the formation of flavan-3-ols such as (+)-catechin and (+)-epicatechin. This kind of compounds are the key subunits for PAs biosynthesis in the last steps.

Over the past decades, it has been commonly accepted that the accumulation of flavonoids is controlled by the structural biosynthetic genes, regulatory MYB transcription factors (TFs), and the MYB-bHLH-WD40 (MBW) complex (Schaart et al., 2013; Xu et al., 2015; Naing and Kim, 2018). In strawberry, *FaMYB10* has been proven to be the major force to regulate the whole anthocyanin biosynthesis pathway (Castillejo et al., 2020). On the contrary, *FaMYB1* and *FaMYB5* were described as putative negative regulator in the strawberry anthocyanin and PAs biosynthesis pathway (Aharoni et al., 2001; Paolocci et al., 2011). Moreover, combined investigations of transcriptome and metabolome data offered a powerful method to understand flavonoids biosynthesis and regulation at both molecular and physiological levels. Various studies have been carried out to identify the candidate genes and regulators for anthocyanins accumulation in strawberry, using the red strawberry and its white mutant by comparative transcriptome analysis (Lin et al., 2018; Zhao et al., 2021). Thus, a lot of TFs, such as WRKY, NAC, and TCP, were also suggested to be involved in the strawberry flavonoid biosynthesis (Lloyd et al., 2017; Sun et al., 2019; Xie et al., 2020). An integrated transcriptome and metabolome analysis was also performed to study the fruit storability of different varieties (Min et al., 2020), and cyanidin metabolism in the pink flower of strawberry (Xue et al., 2019). However, so far, researches about the joint transcriptome and metabolome analysis of the red strawberry and its dark red mutant, especially that contains higher levels of both anthocyanins and PAs content, has not been reported.

Considering of mutational studies contributing to further understanding of the regulatory mechanisms of flavonoid biosynthesis in strawberry, screening mutants with high phytochemical composition is important. Recently, we have identified a new natural mutant (MT) from the cultivation of strawberry ‘Benihoppe,’ which covers the largest cultivated area in China. The fruits of this mutant are in darker red color, also contain higher total phenolic content (TPC), total flavonoid content (TFC), total anthocyanins content (TAC), and proanthocyanins (PAs) concentration comparing with the normal ‘Benihoppe’ (WT). However, the specific components and molecular reasons causing these differences remain unclear. To explore the metabolites' changes and the possible reasons at molecular level during the fruit development of these two specific strawberry samples, an untargeted metabolomics together with transcriptomics method was performed in this study. The results generated here could characterize the metabolic changes in flavonoid pathway during strawberry fruit development and ripening, and also provide a new natural high-flavonoid mutant for future utility in genetic improvement and breeding of horticultural crops.

## Materials and Methods

### Plant Materials

**Figure 1:**
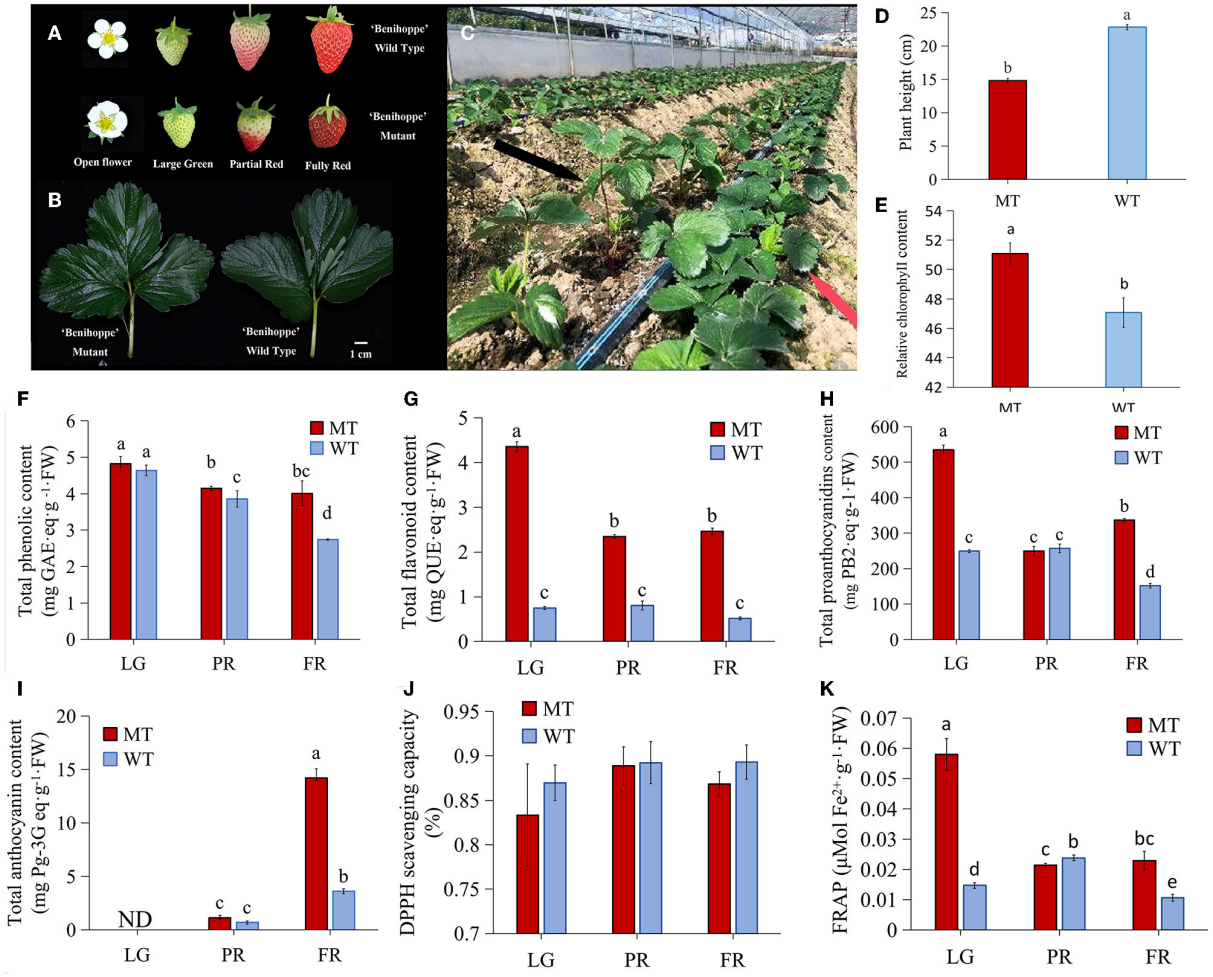
The differences between strawberry ‘Benihoppe’ and its mutant. **(A,B)** The phenotype of flowers, fruits and leaves; **(C)** The phenotype of mutant plants (red arrow) and ‘Benihoppe’ plants (black arrow); **(D,E)** The plant height and relative chlorophyll content; **(F,G)** The total phenolic content and total flavonoid content; **(H,I)** Total anthocyanins content and total proanthocyanidins content; **(J)** The DPPH scavenging capacity; **(K)** FRAP antioxidant capacity of strawberry ‘Benihoppe’ and its mutant at different developmental stages. ND, not detected; LG, large green; PR, partial red; FR, fully red; MT and WT indicated mutant and normal ‘Benihoppe’ separately. The same as below.

The plants of strawberry cultivar ‘Benihoppe’ and its mutant were grown in a greenhouse located in Hanyuan, Ya'an, China. The mutant was a natural mutant identified during the cultivation of ‘Benihoppe’ strawberry. We have observed several natural mutant plants with different phenotypes from the normal ‘Benihoppe’ plants (Figures 1A,B) in year 2018. These mutants' plants were subsequently propagated in a large scale in year 2019. It was found the reproduced mutant plants displayed stably different phenotypes comparing with the normal ‘Benihoppe’ plants. The growth condition was set as 22 ± 2°C, relative humidity 70–90% and a 14/10-h light/dark regime. Since year 2020, this new natural mutant has been widely applied in commercial cultivation (Figure 1C). Three fruit developmental stages were defined as large green (LG), partial red (PR), and fully red (FR) based on days post-anthesis (DPA) and the color of the receptacle. Fruits were collected and subsequently grounded into powder in liquid nitrogen and stored at −80°C until further use. At least three fruits were mixed as one biological replicate, three biological replicates were collected.

### Sample Preparation, Metabolites Extraction, and Metabolome Analysis

Around 100 mg of the grounded each fruit sample were extracted in 500 μl 80% aqueous methanol solution. After centrifugation at 15,000*g*, 4°C for 20 min, the supernatants were filtered and diluted with aqueous methanol. Equal volume supernatants from each sample were mixed and used as quality control sample. A 53% aqueous methanol solution was used as blank control. The quantification was done by Novogene (Beijing, China). Briefly, a Vanquish UHPLC system coupled with an Orbitrap Q ExactiveTMHF-X mass spectrometer (Thermo Fisher, US) was used for UHPLC-MS/MS analyses. Compounds separation was performed by a Hypesil Gold column (100 mm × 2.1 mm, 1.9 μm) using a 17-min linear gradient program. The flow rate was set to 0.2 ml/min. 0.1% formic acid and 5 mM ammonium acetate (pH 9.0) were used as eluent A in the positive and negative polarity mode, methanol was used as eluent B in both polarity mode. The solvent gradient was set as follows: 2% B, 1.5 min; 2–100% B, 12.0 min; 100% B, 14.0 min; 100–2% B, 14.1 min; 2% B, 17 min. The raw data was processed using the Compound Discoverer 3.1 (CD3.1) system. Differential metabolites with the percentage of coefficient of variation (%CV) below 30%, variable importance in the projection (VIP) >1.0, fold changes (FC) ≥2 or ≤ 0.5, and the adjusted *p* ≤ 0.05 were included from the statistical analyses. Six independent replications were included for each sample.

### Ribonucleic Acid Sequencing and Transcriptome Analysis

Total RNA was isolated from three stages (LG, PR, and FR) of WT and MT fruits using the improved CTAB (cetyltrimethylammonium bromide) method (Chen et al., 2012). Libraries were constructed and sequenced by Novogene (Beijing, China). Briefly, mRNA was firstly enriched by the oligo(dT) beads and then fragmented in NEB fragmentation buffer. First- and second-strand cDNAs were synthesized in the presence of random hexamer primers and DNA polymerase I and RNase H. Thereafter, a poly(A) tail and NEBNext adaptors were added to the two stranded cDNAs. Next, cDNAs of size 250–300 base pairs (bp) were selected by using 3 μl of USER Enzyme (NEB, USA), adaptor-ligated cDNA at 37°C for 15 min followed by 5 min at 95°C. Next, PCR amplifications were carried out using Phusion high-fidelity DNA polymerase and universal PCR primers. Then, the quality of the libraries was assessed on an Agilent 2100 bioanalyzer. A total of 18 (three replicates for each stage of MT and WT fruits) libraries were clustered and sequenced (150 bp, pair end) on a Hiseq-2500 platform. The low-quality (*Q* < 20) raw reads were screened by FASTQ software, and the adaptors were trimmed by using trim-galore (v0.6.6). The cleaned reads were mapped onto the reference strawberry genome (v1.0.a2) and quantified using the Hisat 2 and stringtie (v) pipeline (Pertea et al., 2016) with the default parameters. The DESeq 2 (v3.34.1) R package was used to detect the differentially expressed (DE) genes. The genes with log2 transformed FC >1 or < -1, and the adjusted *p* ≤ 0.05 were considered as significantly DE.

### Identification of Potential TFs and Correlation Analysis

The iTAK perl program (v1.7a) was used to search the potential TFs in the RNA-seq data. The conserved domains of each TF sequence were further identified based on the Hidden Markov Model (HMM) profile, which was download from the Pfam protein family database (http://pfam.xfam.org/). The co-expression of DE TFs and transcripts was calculated by R software using the Pearson correlation algorithm; the co-expressed pairs with correlation coefficient *r* > 0.8 and *r* < −0.8 at a *p* < 0.05 were recognized as significantly correlated pairs. The correlation network was visualized by Cytoscape software (v3.4.0).

### Determination of Plant Height and Relative Chlorophyll Content

The height of plants was measured by a Vernier caliper, at least 30 plants of MT and WT were measured and the average values were calculated. The relative chlorophyll content was detected by SPAD-502 chlorophyll meter (Konica Minolta, Japan). The adaxial side of the leaves avoiding the major veins were placed toward the emitting window of the instrument. Each leaf was marked in three up to 10 representative points based on its total leaf area.

### Determination of Total Anthocyanins, PAs, Phenolic, and Flavonoid Content

The total anthocyanin content (TAC) was determined using a pH differential method as previously reported (Ganhão et al., 2019) with slight modification. Briefly, approximately 1.5-g fruit sample was extracted with 15 ml of extraction solution (acetone:methanol:water:acetic acid = 2:2:1:0.5), after a water bath in 40°C, the mixture was centrifuged (8,000*g*, 4°C) for 25 min, and the supernatant was used for measurement. 0.2-M potassium chloride (pH 1.0) and 0.2-M acetate sodium (pH 4.6) were used as buffers for pH difference determination. The absorbance differences at 496 and 700 nm were taken to calculate the TAC; TAC was expressed as microgram of pelargonidin 3-glucoside (Pg3G) equivalents per g of FW fruits.

The total soluble PAs content was measured by an improved DMAC (4-dimethylaminocinnamaldehyde) method according to previous report (Prior et al., 2010). In brief, around 1.5-g plant samples were extracted into 2% (v/v) acetic acid. The extracts were vortexed and sonicated for 1 h at room temperature. Thereafter, the mixture was centrifuged for 20 min at 10,000*g*. The supernatant was collected and analyzed by a 96-well plate through an UV spectrophotometry at 640 nm. Total soluble PAs were represented by mg procyanidin B2 equivalents per g of fresh weight (FW) of fruits.

For TPC and TFC determination, about 1-g fruit samples were extracted in 1% HCL solution prepared in methanol for 2 h at room temperature. After centrifuged at 5,000 rpm, the supernatants were used for analysis.

The TPC was determined with the Folin–Ciocalteu's assay according to the previous study (Ganhão et al., 2019). In the procedure, extracts from plants (~1 mg) were mixed with 1.5 ml Folin- Ciocalteu's reagent (FCR) diluted 1:10 (v/v). 0.8 ml of 7% (w/v) Na_2_CO_3_ solution was added after the mixture was stand for 10 min. The final mixture was heated in a 40°C water bath for 20 min, and cooled in an ice bath. Absorbance of sample was measured against the blank at 760 nm using a spectrophotometer. Gallic acid was used as standard, and TPC was represented as gallic acid equivalent (GAE) per g of FW of fruits.

The TFC was determined by aluminum chloride method using quercetin as a standard based on a previous study (Parra-Palma et al., 2017). In brief, the 5-mg fruits extracts were diluted with 5 ml distilled water, and 0.3 ml of 5% NaNO_2_ was added; after 5 min, 0.6 ml of 10% AlCl_3_ was added and stand for 5 min; at last, 2 ml of 1.0 M NaOH was added. The absorbance of the reaction mixture was measured at 510 nm using a spectrophotometer. Total flavonoid content was described as quercetin equivalents (QUE mg/g FW). All extraction experiments were repeated 3 times independently, three readings were taken each time for each sample, and the results were calculated average values.

### Antioxidant Capacity Assays

Radical scavenging ability was detected by DPPH assay as previously reported (Ganhão et al., 2019). Simply, 50 μl of sample/standard was added into 150 μl of DPPH solution (150 μM) for reacting 1 h in dark at room temperature. Then, the absorbance was detected at 517 nm using a microplate reader. The percentage of inhibition was expressed as radical scavenging activity.

Ferric-reducing antioxidant power (FRAP) was determined based on the previous described method with slight modification (Ganhão et al., 2019). A 10:1:1 ratio of 0.3-M acetate buffer (pH 3.6), 10-mM of 2,4,6-tripyridyl-*s*-triazine (TPTZ) in 40 mM HCL, and 20 mM ferric solution using FeCl_3_ were freshly mixed to be the final working FRAP reagent. In the procedure, 2 μl of sample was reacted to 198 μl of FRAP reagent at 37°C in dark for 30 min, and the increase in absorbance at 593 nm was recorded. A standard curve was made using 0.1–1.0-mM FeSO_4_ solution, the results were expressed in μM of FeSO_4_ per 100 g of FW fruits. In DPPH scavenging activity and FRAP methodologies, three independent reactions from each sample were measured.

### Validation of Selected Anthocyanins and PAs by HPLC Method

The anthocyanins (cyanidin 3-glucoside, pelargonidin 3-glucoside, pelargonin chloride, cyanidin 3-*O*-rutinoside, and cyanidin chloride) and the PAs (procyanidin B1 and catechin) were selected to validate the metabolome results by HPLC method. As previously described by Donno et al. (2013), and Yonekura and Tamura (2019), anthocyanins and PAs were extracted using 1% HCL in methanol solution and 30% methanol, respectively. They were subsequently detected by a Silgreen ODS C18 column using Agilent HPLC system with a DAD detector at 510 and 210 nm. The anthocyanins, listed as follows, were detected using a linear gradient eluent program: 5% formic acid in water as eluent A and methanol as eluent B, 100-0% A in B was used for 20 min, followed by 100% B for 5 min. However, the PAs were detected by an isocratic eluent procedure using acetonitrile/water 12:88 (v/v) with 0.1% formic acid as mobile phase for 16 min. A 10-μl sample was injected, flow rate was set to 1 ml/min. The concentration of anthocyanins and PAs were quantified by comparing with the corresponding external standards, all HPLC-grade standards were purchased from Sigma (USA). Experiments were independently repeated three times.

### Real-Time Quantitative PCR

A real-time quantitative PCR (qPCR) assay was performed to validate the expression levels of various selected genes in strawberry fruit samples. A 10-μl reaction system was established including 1-μL of cDNA template, 1 μl of gene specific primer pairs (Supplementary Table S7), and 5 μl of TB Green Premix Ex Taq II (TaKaRa, Dalian, China). Three-step PCR reactions were done with a denaturation step at 94°C for 30 s, an annealing step at 58°C for 10 s, and an extension at 72°C for 10 s. The total reaction circle was set to 40. The *FaActin2* gene (LOC101313255) was selected as an internal control. The relative expression levels of the detected genes were calculated by the 2^−Δ*ΔCt*^ method. Three wells of each sample were done as three technological replicates, and three independent biological replicates were done for all the qPCR reactions.

### Statistical Analysis

The data was analyzed using IBM SPSS Statistics software (v25.0). The results were expressed as mean ± standard deviation (SD). A *p* ≤ 0.05 was considered a statistically significant difference (Turkey's multiple range test).

## Results

### Phenotypic Variation of Identified Mutant

A natural mutant was identified from the strawberry cultivar ‘Benihoppe’ cultivation process (cultivated in Hanyuan, Ya'an, China), which exhibited visible phenotype changes. First of all, the open flowers of WT were fully expanded, while the MT flowers at the same developmental stage were not (Figure 1A). Besides, WT fruits showed bright red color while the MT fruits exhibited dark red although they were at the same developmental stage (Figure 1A). The size of the fully expanded leaves was not obviously different (Figure 1B), but the height of MT plants was significantly reduced comparing with the WT plants, and thus the MT plants displayed dwarf phenotype (Figures 1C,D). Furthermore, the leaves from MT plants contained higher relative chlorophyll content than which from WT plants (Figure 1E). More important, the fruits of MT accumulated apparently more TPC and TFC during the ripening process than the WT fruits, which were up to 2-folds and 4-folds higher in MT than that in WT fruits at FR stage, respectively (Figures 1F,G). Additionally, the dynamic changes of TPC during the developmental stages suggested a gradually decrease in WT and MT fruits (Figure 1F). The TFC was at the highest level in LG stage, and decreased at PR stage, then kept at a relative stable level in FR stage in MT (Figure 1G). However, the TFC did not show significant changes from LG to PR stage in WT fruits (Figure 1G). Likewise, the content of PAs in MT fruits was detected at the maximum level at LG stage, and sharply decreased at PR stage, while it increased obviously at FR stage showing 2 times higher level than that in WT fruits (Figure 1H). By contrast, the TAC showed an increasing pattern during fruit development and ripening in both MT and WT fruits. Notably, the MT fruits accumulated much more anthocyanins (over 3-folds) than the WT fruits (Figure 1I). Furthermore, the results of antioxidant activity measurement showed that, there was no obvious difference in 1,1-diphenyl-2-picryhydrazyl (DPPH) scavenging activity between MT and WT fruits (Figure 1J). However, a significant higher level of FRAP at LG and FR stages, while a lower FRAP level at the PR stage in MT than that in WT fruits was observed (Figure 1K). These results suggested that the MT fruits have clear different phenotypes from the WT fruits, and contain higher levels of health-attributing phenolic compounds.

### Overall Analysis of Metabolome Data

**Figure 2:**
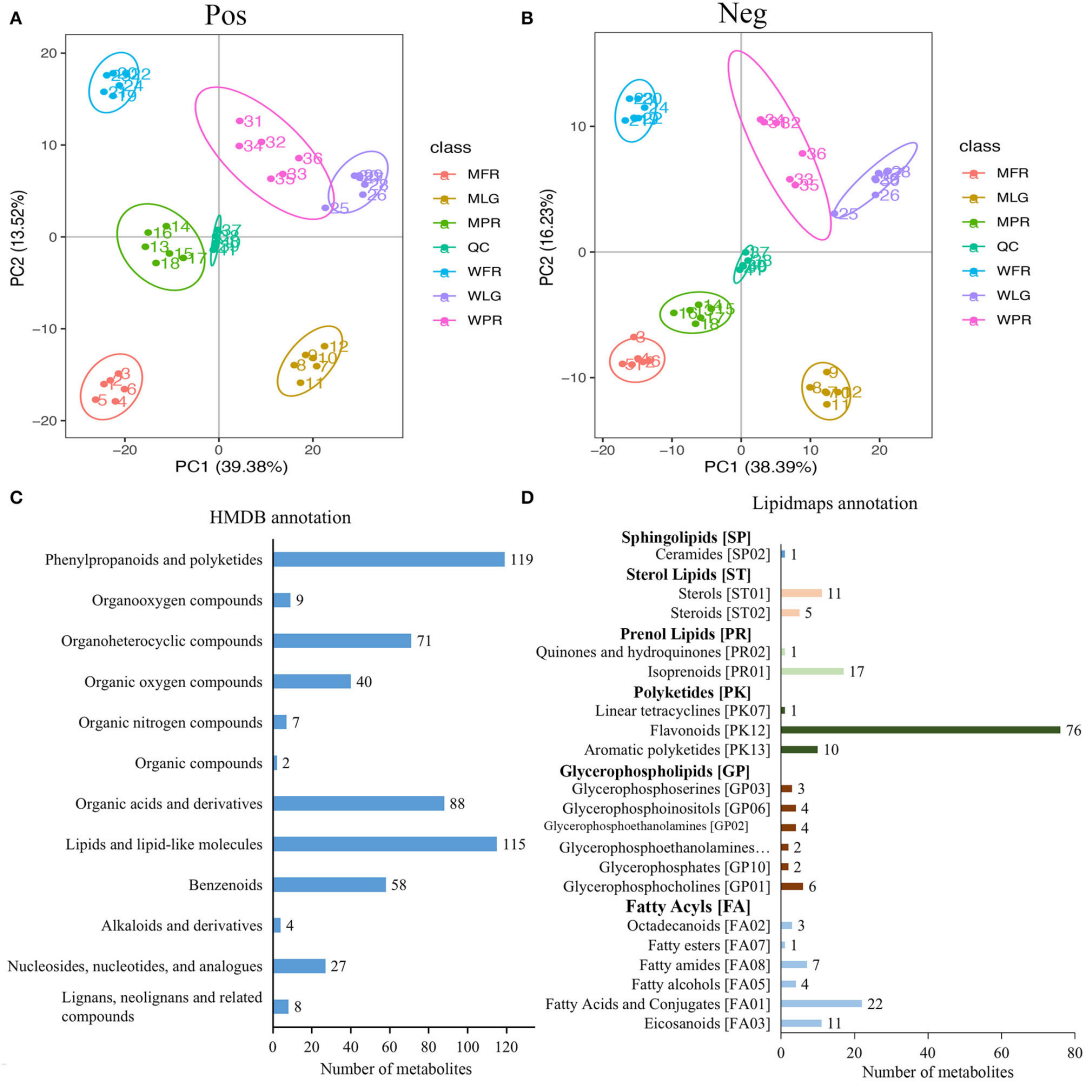
Overall analysis of metabolome data of strawberry samples at different developmental stages. **(A,B)** PCA plot of metabolome data in positive and negative ion modes separately; **(C,D)** indicated the annotation of identified metabolites in HMDB and Lipidmaps databases. WLG, WPR, WFR indicated large green, partial red, and fully red stage of normal ‘Benihoppe’ fruits, MLG, MPR, MFR represented large green, partial red, and fully red stage of mutant fruits the same below.

The metabolic profiling of WT and MT in different developmental stages (six replications in each) were obtained using ultra-high-performance liquid chromatography/quadrupole time-of-flight mass spectrometry (UPLC/Q-TOF-MS/MS) method. From the principal component analysis (PCA) plot of the data in positive and negative ion modes, we could see that 41 samples (including 5 quality control samples and 36 experimental samples) were significantly separated into 7 clusters corresponding to three different developmental stages in each material. Then, PC1 and PC2 explained around 52 and 54% of total variation for positive and negative ion samples, respectively (Figures 2A,B). In addition, the quality control (QC) samples made up of the mixture of all experimental samples were clearly separated on the PCA plot, indicating the high diversity among the samples.

In total, 1,375 metabolites were detected in strawberry samples at three developmental stages in two varieties. All the identified metabolites were furtherly annotated using the HMDB and Lipidmaps databases. As suggested, 548 metabolites were classified into 12 categories in the HMDB database (Figure 2C), the largest types were phenylprpanoids and polyketides (119 metabolites), followed by lipids and lipid-like molecules (115 metabolites), organic acids and derivatives (88 metabolites), and organoheterocycle compounds (71 metabolites). According to the Lipidmaps dataset (Figure 2D), the glycerophospholipids and fatty acyls types included the most categories (six categories), while the polyketides type consisted of the most metabolites (87 metabolites).

### Differentially Accumulated Flavonoids Between WT and MT Fruits

**Figure 3:**
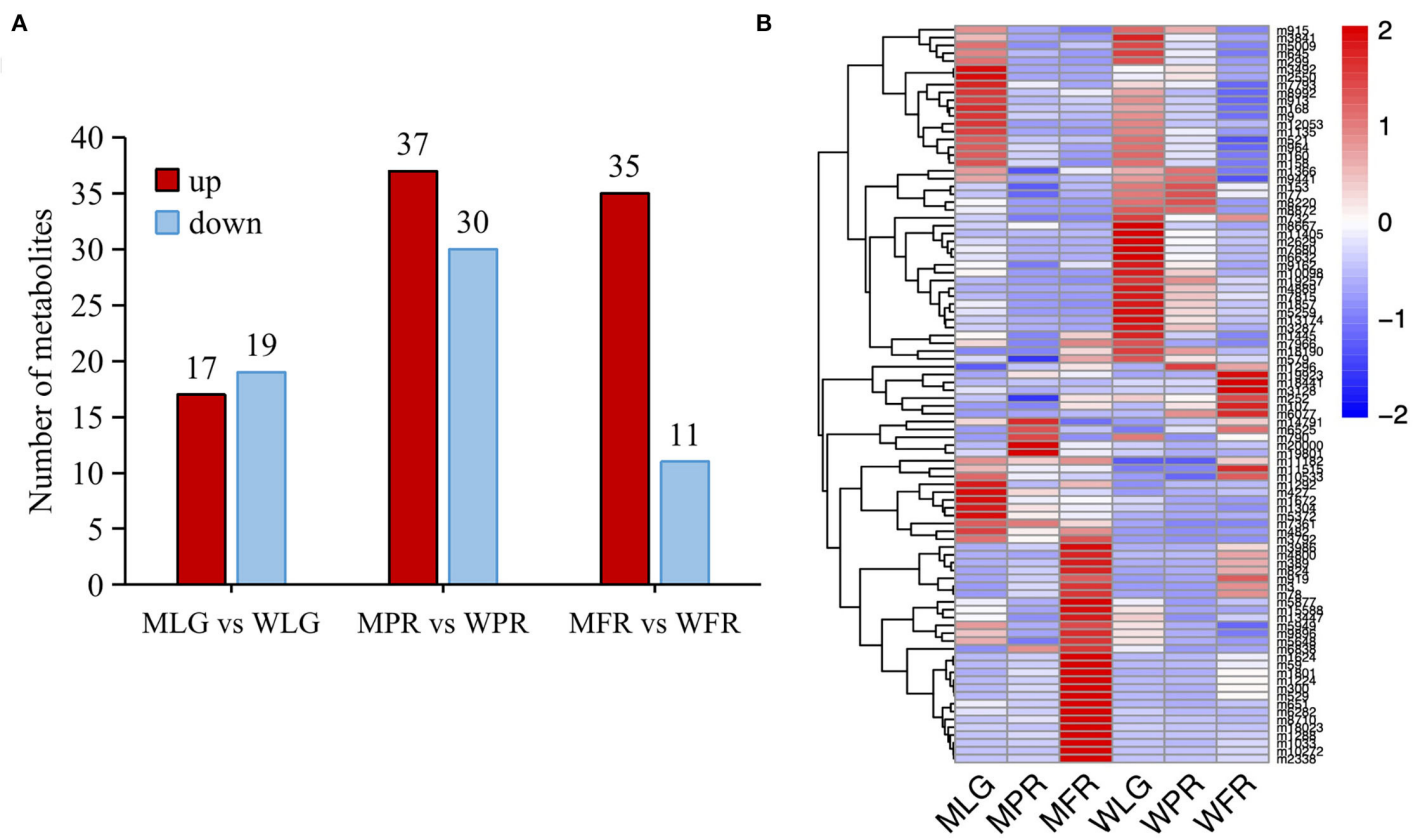
The differentially accumulated flavonoids in WT and MT samples. **(A)** Comparison of up- and down-regulated flavonoids of different developmental stages in two varieties. **(B)** Heatmap showing the intensities of all the DA flavonoids. The heatmap was represented by the z-score values of the mean peak area of 6 biological replicates.

In total, there were 113 flavonoids detected in the strawberry samples (Supplementary Table S1). Based on FC≥ 2 or ≤0.5 and VIP≥ 1, 36, 67, and 46 compounds were identified as differentially accumulated (DA) metabolites between MT and WT at LG, PR, and FR stages separately (Figure 3A, Supplementary Table S1). In the MLG *vs*. WLG group, the up- and down-regulated metabolites numbers were similar (17 up-regulated and 19 down-regulated), this was also observed in the group of MPR *vs*. WPR (37 up-regulated and 30 down-regulated). However, in the MFR *vs*. WFR group, most (35 out of 46) of the DA flavonoids were up-regulated (Figure 3A). The intensities of the DA flavonoids were normalized and analyzed by hierarchical clustering analysis. As shown in Figure 3B, the DA flavonoids abundance showed a clear grouping pattern, all of the DA flavonoids were clustered into two large groups. Group 1 exhibited highly accumulation of metabolites in LG stage of WT fruits, while group 2 showed that most metabolites were highly accumulated in the FR stage of mutant.

**Figure 4:**
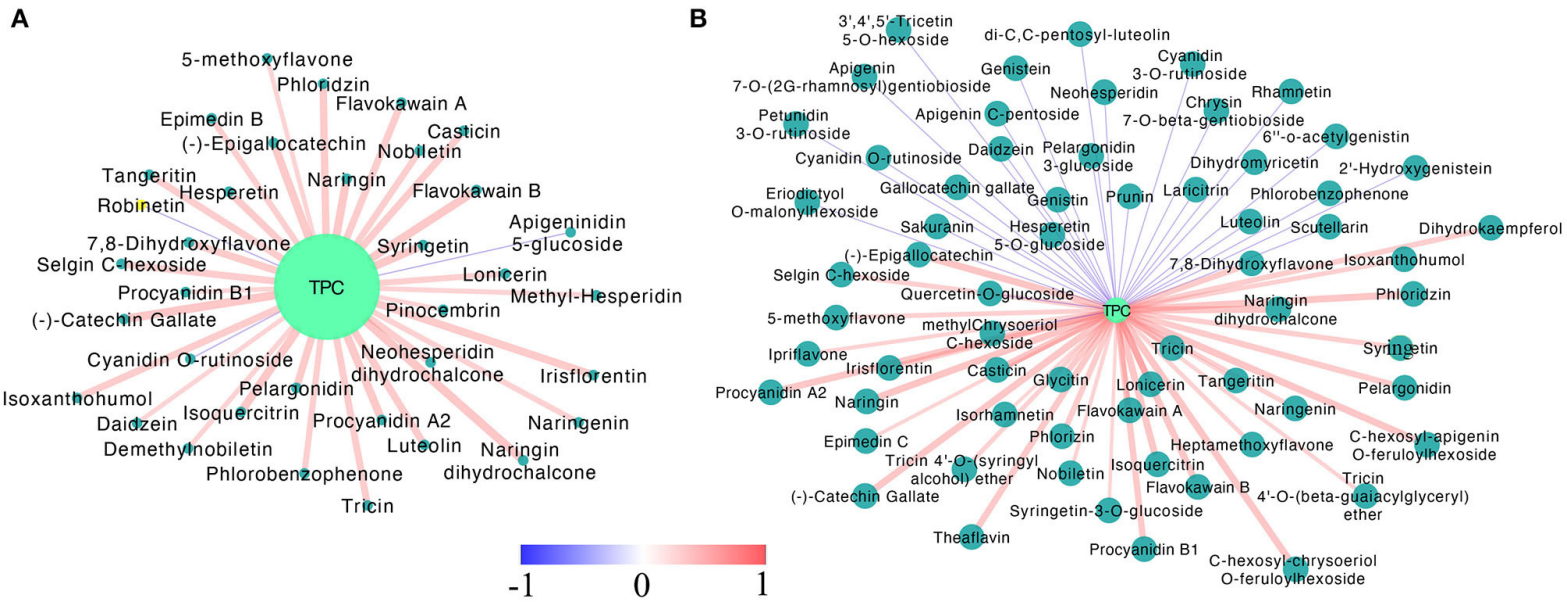
The Pearson correlation analysis of differentially accumulated flavonoids and total phenolic contents in MT **(A)** and WT **(B)** fruits during fruit development and ripening. Only the pairwise with *p*-values ≤ 0.05 were showed. The dark and light blue circles indicated differentially accumulated flavonoids and the total phenolic content, respectively. The red and blue lines suggested positive and negative correlation, color scale, and the line width indicated the coefficient value, the wider the line, the larger the *r* value.

To explore what flavonoids contributed to the relative stable TPC in the late developmental stage (PR to FR) of the MT fruits, the Pearson correlation analysis of the DA flavonoids and the TPC was conducted (Figure 4, Supplementary Table S2). As the results showed, in the MT fruits, 34 DA flavonoids were found significantly positively correlated with TPC, while only two metabolites (cyanidin *O*-rutinoside and apigenindin 5-glucose) showed negative correlation with TPC (Figure 4A). However, 27 and 36 metabolites were found significantly negatively and positively correlated with TPC in WT fruits, respectively. Notably, the PAs including procyanidin A2, procyanidin B1, (–)-catechin gallate and (–)-epigallocatechin and the non-glycosylated pelargonidin showed significant positive correlation with TPC in both WT and MT fruits. By contrast, the glycosides formed anthocyanins including cyanidin *O*-rutinoside, petunidin 3-*O*-rutinoside and cyanidin 3-*O*-rutinoside were highly negatively correlated with TPC in WT fruits. The cyanidin *O*-rutinoside was also found negatively correlated with TPC in MT fruits. These results revealed that the PAs rather than anthocyanins contributed most to the TPC in our detected strawberry samples, leading to the gradually decrease trend of TPC during the WT fruit development while a stable trend in the late fruit development stage of MT fruit.

### Differential Analysis of Anthocyanins and PAs in the Detected Strawberry Samples

**Figure 5:**
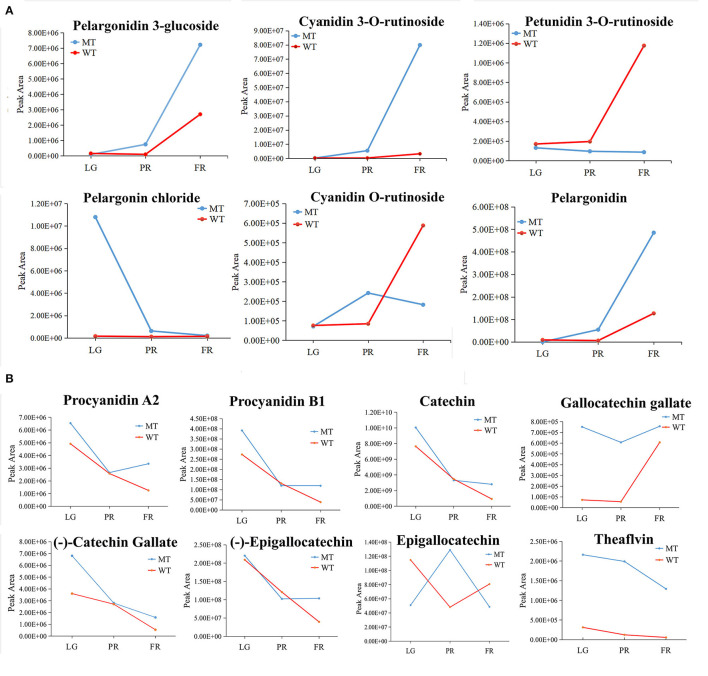
The change patterns of individual differentially accumulated anthocyanins **(A)** and PAs **(B)** during the fruit development in MT and WT samples. LG, PR, and FR indicated the large green, partial red, and fully red stages.

A total of six anthocyanins and eight PAs were found differentially accumulated in mutant fruits comparing with WT fruits. Among the anthocyanins, the accumulation of pelargonidin 3-glucoside, cyanidin 3-*O*-rutinoside and pelargonidin gradually increased during fruit development in both MT and WT fruits. The cyanidin 3-*O*-rutinoside was more than 24-folds higher in MT than WT at FR stage (Figure 5A). On the contrary, the petunidin 3-*O*-rutinoside maintained at a relative stable level in MT, while increased gradually in WT during fruit development and ripening (Figure 5A). At FR stage, petunidin 3-*O*-rutinoside reached to the highest level in MT, showing 3-folds higher than that in WT fruits (Figure 5A). Moreover, the pelargonin chloride decreased gradually but relative stable trend during the fruit development of MT and WT, respectively, which there resulted a significant upregulation in MT at LG stage. In addition, the cyanidin *O*-rutinoside showed opposite change trends with a firstly increase from LG to PR and thereafter a decrease until FR stage in MT, while a stable trend from LG to PR and subsequently a sharp increase from PR to FR stage in WT (Figure 5A).

In the terms of PAs, six out of the eight differentially accumulated PAs including *p*-procyanidin A2 and B1, gallocatechin gallate, (–)-epigallocatechin, catechin and (–)-catechin gallate were decreased in content during the fruit development in WT fruits. However, they were initially dropped and then rose or maintained at stable levels from PR stage to FR stage in MT, and thus lead to an upregulation of PAs in MT at FR stage comparing to WT. The trends opposite to these six PAs were observed for epigallocatechin in MT, and gallocatechin gallate in WT (Figure 5B). Specifically, although the change patterns of theaflavin and gallocatechin gallate were similar in WT and MT during fruit development, the levels of them were largely up-regulated in MT.

### Validation of Selected Anthocyanins and PAs Content

**Figure 6:**
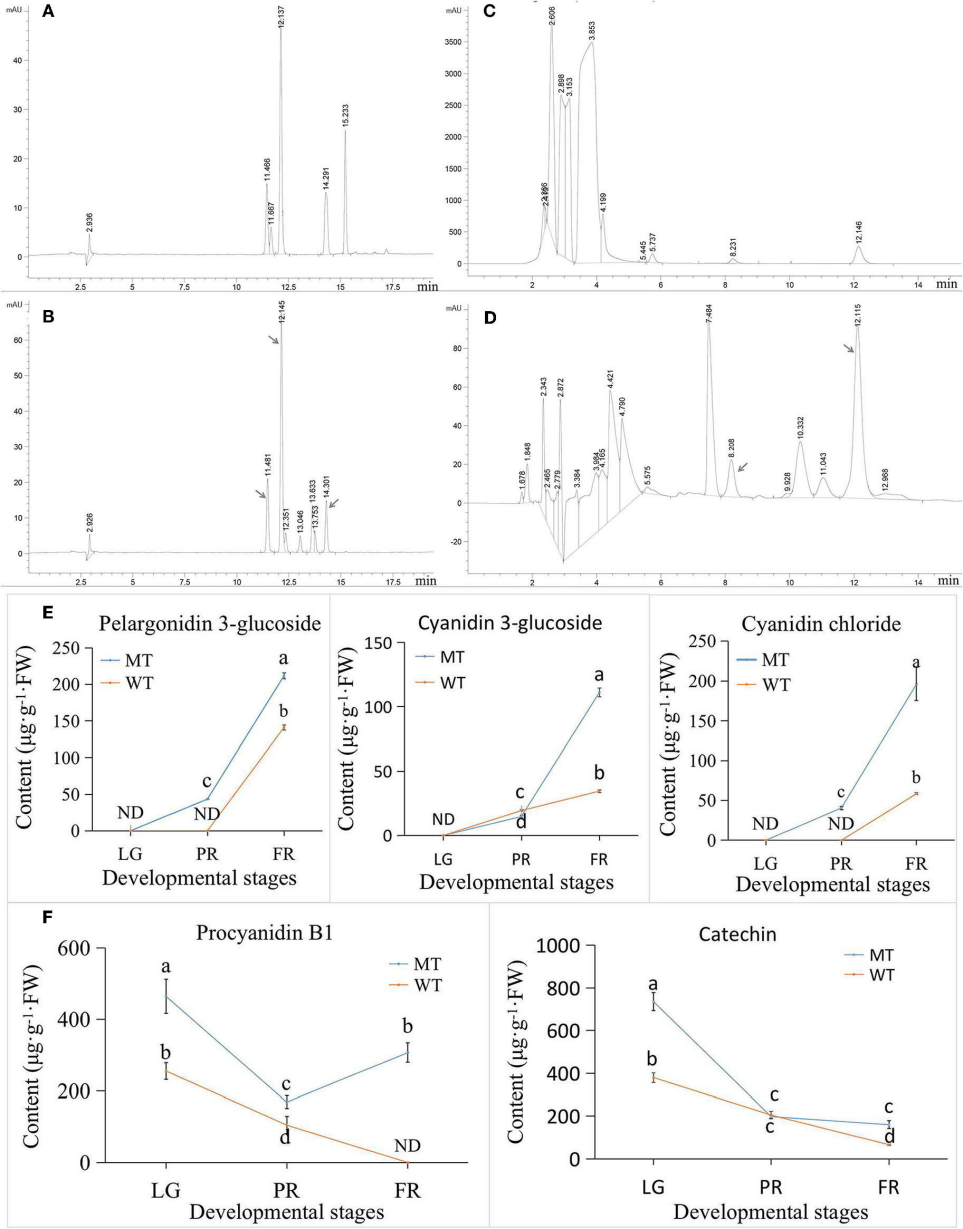
The HPLC analysis of selected anthocyanins and PAs in strawberry. **(A)** HPLC peaks of five anthocyanins standards. Five peaks with retention time of around 11.4, 11.6, 12.1, 14.3, and 15.2 min indicated cyanidin 3-glucoside, cyanidin 3-*O*-rutinoside, pelargonidin 3-glucoside, cyanidin chloride, and pelargonidin chloride, respectively. **(B)** The HPLC analysis of anthocyanins in strawberry samples. The peaks of corresponding anthocyanins were pointed by arrows. **(C)** The HPLC peaks of 2 PAs standards. The peaks at retention time 8.2 and 12.1 min were procyanidin B1 and catechin, respectively. **(D)** The HPLC analysis of PAs in strawberry samples. The peaks of corresponding PAs were pointed by arrows. **(E)** The content of HPLC detected anthocyanins. **(F)** The content of HPLC detected PAs. ND, not detected; MT, mutant fruits; WT, normal ‘Benihoppe’ fruits; LG, large green stage; PR, partial red stage; FR, fully red stage.

To validate the results of metabolome, seven representative flavonoids (including five anthocyanins and two PAs) were selected to detect using HPLC method (Figures 6A,B). However, only five of them were detected in our results (Figures 6C,D). Notably, the cyanidin 3-O-rutinoside, suggested as the largest changed anthocyanin in MT comparing with WT (Figure 5A) by metabolome analysis, was not detected by HPLC method. Likewise, the pelargonin chloride was also not detected by HPLC. Probably because the concentration of those two compounds in strawberry was too low to be detected by HPLC. By contrast, the cyanidin chloride was not detected by metabolome analysis (Supplementary Table S1), while it was detected by HPLC (Figure 6E), which might be caused by the different methods. The content of cyanidin chloride was significant higher in MT than WT. The other two selected anthocyanins including cyanidin 3-glucoside and pelargonidin 3-glucoside were also detected in higher levels in the MT fruits than that in WT fruits, with an exception of cyanidin 3-glucoside at PR stage (Figure 6E). In the terms of PAs, the changes of procyanidin B1 and catechin content detected by HPLC (Figure 6F) exhibited same trend with the results detected by metabolome (Figure 5B). They gradually decreased during the ripening process in WT. However, in MT, they decreased only from LG to PR stage and followed by a slightly increase from PR to FR stage (Figure 6F). Despite of the change of exact individual compound, the HPLC results revealed higher anthocyanins especially cyanidin-based types, as well as PAs in MT than WT, which confirmed the validity of metabolome analysis. Transcriptome analysis and differentially expressed genes involved in flavonoid metabolism.

### Transcriptome Analysis and Differentially Expressed Genes Involved in Flavonoid Metabolism

**Figure 7:**
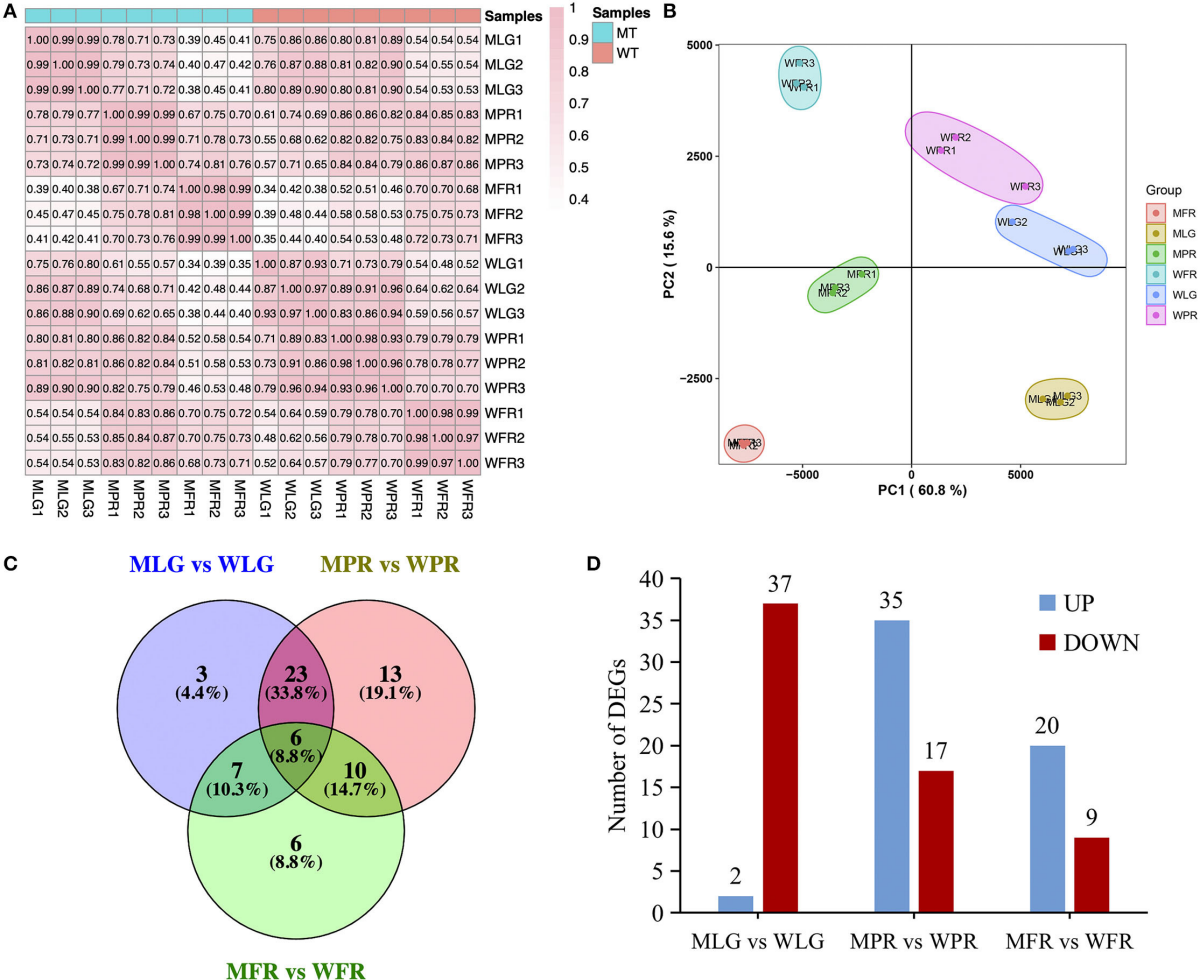
Comprehensive analysis of transcriptome data. **(A)** Pearson correlation analysis of samples used for RNA-seq. **(B)** PCA plot for the transcriptome data. **(C)** Venn diagrams showed the overlap of DE FRG in MT and WT fruits. **(D)** The numbers of significantly up-regulated and down-regulated genes in the different developmental stages of MT comparing with WT. LG, large green; PR, partial red; FR, fully red.

Also, the transcriptome profiling of the same samples with metabolome analysis (three replications in each) was acquired by RNA-sequencing (RNA-seq). The samples correlation analysis (Figure 7A) and PCA analysis (Figure 7B) suggested that the transcriptomics data were clearly clustered according to the different developmental stages of WT and MT, respectively. To understand the potential molecular mechanism of the altered flavonoids in the MT fruit, the biosynthetic genes involved in flavonoids pathway were analyzed using transcriptome analysis. According to the results, a total of 108 genes were identified as flavonoids-related genes (FRG, Supplementary Table S3) based on annotation. Among them, 39, 52, and 29 FRG were differentially expressed (DE) (|log_2_FC| ≥1, padj ≤ 0.05) at LG, PR and FR stage respectively in MT comparing to WT (Figure 7C, Supplementary Table S3). Specifically, most FRG were down-regulated in MLG *vs*. WLG group, while up-regulated in MPR *vs*. WPR and MFR *vs*. WFR groups, with 2, 35, and 20 FRG were up-regulated, while 37, 35, and 20 FRG were down-regulated corresponding to LG, PR, and FR stages in MT comparing with WT (Figure 7D).

**Figure 8:**
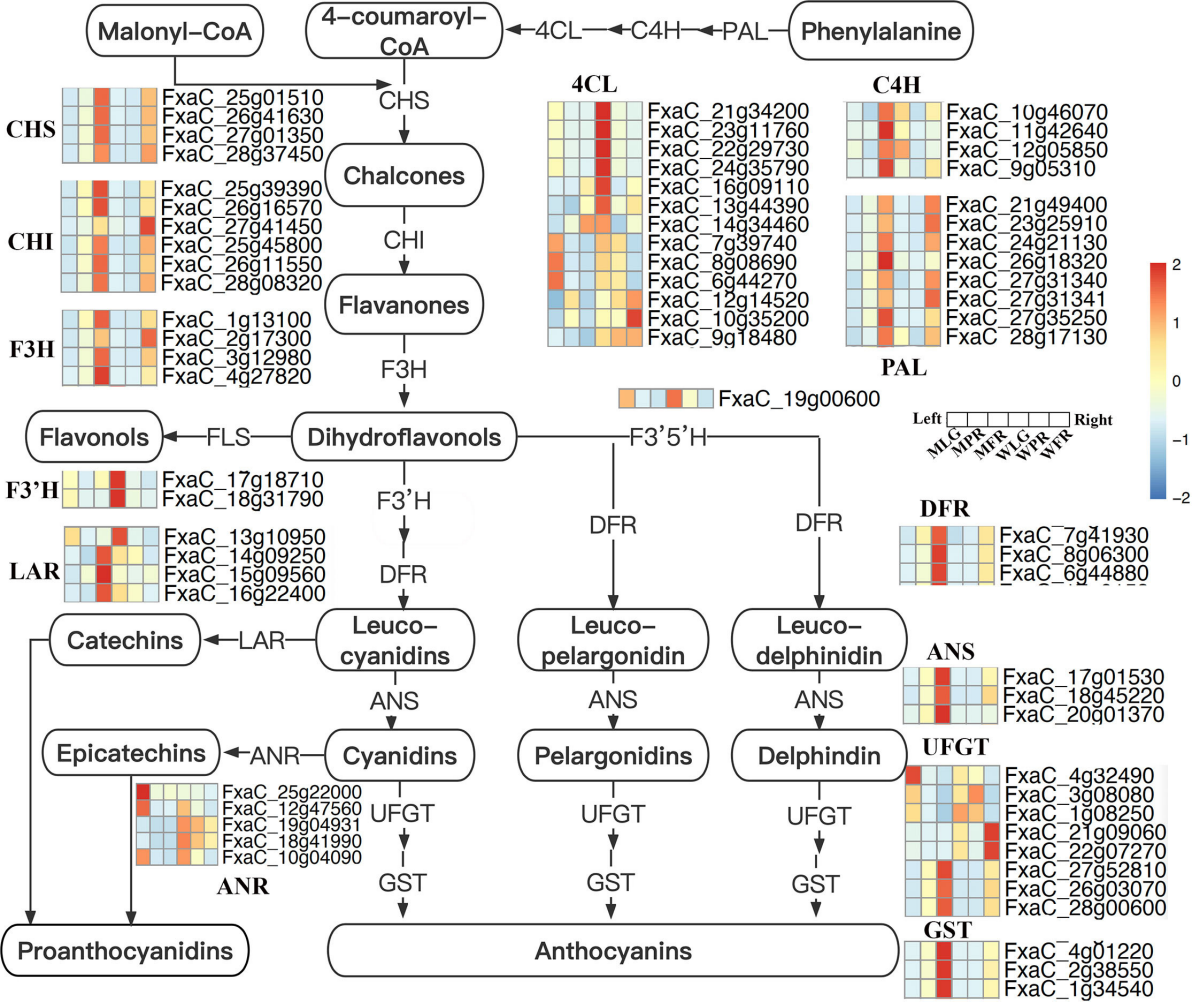
Flavonoids biosynthesis pathway in strawberry. Genes expression were scaled using *z*-score of mean FPKM value from three biological replicates, represented by heatmaps at the side of each step. The cells from left to right indicated the large green, partial red, fully red stages of mutant, and normal ‘Benihoppe,’ respectively. The full names of each gene were shown in Supplementary Table S3.

The DE FRG were mapped to the flavonoids biosynthesis pathway (Figure 8), which showed the genes encoding PAL, C4H, and CHS involved in the early steps were significantly up-regulated at PR and FR stages in MT comparing with that in WT fruits. Also, the expression of genes encoding 4CL, CHI, F3H, F3'H, ANS, DFR, and UFGT were both enhanced or reduced in MT by comparing to WT fruits. In particular, the transcription level of one gene (FxaC_17g18710) encoding F3'H was largely up-regulated at FR stage, the expression of gene (FxaC_19g00600) encoding F3'5'H were significantly down-regulated at PR stage in MT. In addition, four genes (FxaC_13g10950, FxaC_14g09250, FxaC_15g09560, and FxaC_16g22400) encoding LAR were largely up-regulated in MT at FR stage (Figure 8), which probably caused the up-regulation of PAs in MT at FR stage.

The expression of FRG was validated by qPCR method, five DE FRG were randomly selected for qPCR analysis in the MT and WT strawberry (Supplementary Figure S1). Despite the exact change value, high congruence of the relative expression from qPCR results and the FPKM FC from RNA-seq results was found, which confirmed the validity of the RNA-seq data.

### Conjoint Analysis of the Transcriptome and Metabolome Data

**Figure 9:**
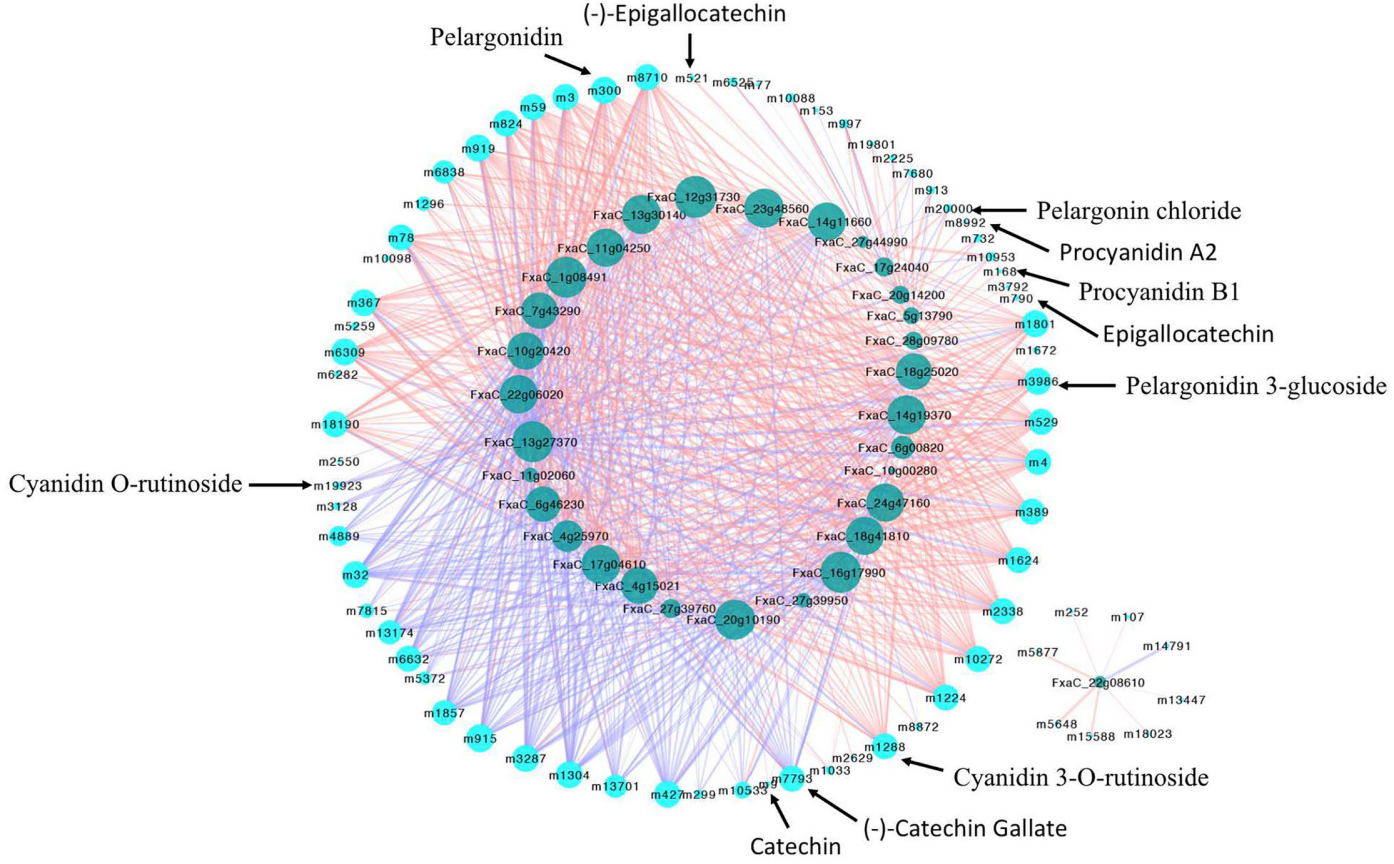
Network showing the correlation between up-regulated TFs and metabolites during the fruit development and ripening of mutant. Genes-metabolites correlation pairs with *p* < 0.05 were showed. The light and dark blue nodes represented metabolites and TFs, respectively. Blue and pink lines represented negative and positive correlation, respectively.

Since the flavonoid biosynthesis pathway is largely regulated by transcription factors (TFs), the DE TFs were identified. There were 1,874 DE TFs identified by comparing MT to WT fruits at each stage (Supplementary Table S4). Among them, 323 DE TFs belonging to MYB, bHLH, bZIP, WRKY, AUX/IAA, NAC, TCP, MADS, GRAS were chosen to conduct the correlation analysis with genes and metabolites. As results (Supplementary Table S5), 128 and 2741 significantly correlated pairs (|PCC| *r* > 0.8, *p* < 0.05) between structural genes and metabolites, TFs, and genes were observed. Besides the well-known TF MYB10 (FxaC_2g30690), several TFs belonging to bZIP, NAC, MADS, and GRAS. etc. families were found to be highly correlated with flavonoids biosynthetic genes, such as *ANR, PAL, 4CL, DFR*, and *ANS*, etc. Furthermore, considering of the fact that most flavonoids were up-regulated in mutant, the DA flavonoids and 30 up-regulated TFs in mutant with high expression levels were selected for correlation analysis (Figure 9, Supplementary Table S6). It was found that most DA anthocyanins including pelargonidin 3-glucoside, pelargonidin, pelargonin chloride, and cyanidin 3-*O*-rutinoside were positively correlated with various up-regulated TFs. Four PAs were only correlated with one or two TFs. Noticeably, three of them (procyanidin B1, (–)-epigallocatechin and epigallocatechin) were found correlated with the zinc finger protein C3H (FxaC_28g09780), other two PAs namely procyanidin A2 and (–)-epigallocatechin exhibited high positively correlation of one MADS box protein (FxaC_20g14200). This result indicated that the up-regulation of these PAs in mutant might be caused by the up-regulation of these TFs. By contrast, two Pas, namely, (–)-catechin gallate and catechin was found negatively with various TFs, which were most annotated as ERF TF types (Figure 9, Supplementary Table S6).

## Discussion

### The Mutant Tends to Accumulate Higher Levels of Flavonoids Throughout Fruit Ripening

Strawberries are enriched in polyphenols, mainly represented by flavonoids. The roles of polyphenols contributing to strawberry fruit taste, appearance, and human health have been well characterized (Ozdal et al., 2016; Toma et al., 2020). Therefore, selection of high nutritional quality with high level of TPC is an important target for strawberry breeding. In this study, we have identified a new natural mutant with dark red appearance of fruits from cultivation process of ‘Benihoppe.’ The higher TPC, TFC, TAC and PAs content (Figure 1) indicated that this mutant has higher antioxidant properties and could be used for subsequent breeding programs.

The difference of TPC depends on genotype and the maturity stage of, strawberry fruit. With the strawberry fruit ripening, both increased and decreased trend in TPC were observed (Pineli et al., 2011; Mahmood et al., 2012). One explanation for the increased TPC is probably due to the gradual increase in fruit ripening process of anthocyanins, which is the most abundant polyphenolic compounds and account for around 41% of the TPC (Aaby et al., 2012). In this study, the anthocyanins content increased with fruits ripen as expected (Figure 1I), while the TPC decreased gradually in WT coordinated with fruit ripening from LG to FR stage, but it was maintained at a relative stable level from PR to FR stage in MT (Figure 1F). Hence, we speculated that there might be other compounds account more for the TPC than anthocyanins, which lead to a decrease of TPC in the detected ‘Benihoppe’ strawberry and its MT. Generally, the PAs contents decreased when strawberry fruit ripen (Zhang et al., 2018). In our results, the PAs showed a trend of slight increase first and then decrease during the fruit ripening process in WT, while the PAs in MT showed the opposite trend and obvious increased from PR to FR stage, suggesting a different accumulation pattern of PAs in strawberry ‘Benihoppe’ and its mutant. Moreover, a significant positively correlation between PAs and TPC during fruit development (Figure 4) would imply that the PAs accounted most to TPC in ‘Benihoppe’ and its MT. This result was similar to the strawberry fruits grown in Trentinoo, in which PAs represented between 54.8 and 77.4% of polyphenolic compounds (Gasperotti et al., 2015).

### Anthocyanins Identified in Strawberry During Fruit Development and Ripening

Anthocyanins are considered as an important sensory trait influencing consumers' choices. The most common anthocyanidins (aglycones) are suggested as pelargonidin, cyanidin, peonidin, delphinidin, petunidin and malvidin (Liu et al., 2018). In strawberry, pelargonidin, and cyanidin derivatives are the major anthocyanidins (Dzhanfezova et al., 2020). Besides, the derivatives of peonidins and malvidins were also proved to present in trace amounts, while the petunidin and delphinidin were not detected in strawberry (Cerezo et al., 2010; Corona et al., 2011). In this study, we have detected not only the pelargonidin and cyanidin, but also a 3-rutinoside of petunidin for the first time in ‘Benihoppe’ and its MT (Supplementary Table S1). These results indicated that the petunidins are also involved in the coloration of specific strawberry variety. Due to the instability of anthocyanidins (aglycones), they are bound to glycosides to form stable anthocyanins (Khoo et al., 2017). Glucoside and rutinoside are the most common two glycosides in strawberry (Da Silva et al., 2007), which were also detected in ‘Benihoppe’ fruit and its MT in our results (Supplementary Table S1). Besides, various acylated anthocyanins such as pelargonidin 3-acetylglucodise and pelargonidin succinylglucoside, which confer higher stability than other anthocyanins (Jokioja et al., 2021), were also reported in several strawberry cultivars (Da Silva et al., 2007). However, no acylated anthocyanins were detected in this study (Supplementary Table S1), indicating the acylation of glycosyl moieties of anthocyanins might not occurred in the detected strawberry samples frequently during fruit development and ripening.

Anthocyanins classes and concentrations determine the fruit color. Normally, fruits with a preponderance of pelargonidins exhibit orange-red color, deep red color with cyanidins, as opposed to blue-red or purple color with delphinidins (Pritts, 2003; Khoo et al., 2017). The peonidins derived from cyanidin contribute to purplish–red, while petunidins and malvidins derived from delphinidins contribute to dark red or purple color (Pritts, 2003; Khoo et al., 2017). Also, high anthocyanins contents are in tend to make plants darker in color. In this study, the TAC was higher in MT than that in WT (Figure 1I). Furthermore, among all the up-regulated anthocyanins in MT, the most marked changes were cyanidin 3-*O*-rutinoside with more than 24-folds higher in MT than WT, followed by pelargonidin and pelargonidin 3-glucoside with around 8 times higher in MT than WT (Figure 5A). These results suggested although these anthocyanins components together contributed to the darker red color appearance of MT fruits, while the cyanidin 3-*O*-rutinoside might function as the primary one. This is similar to the strawberry variety Malwina (Oliveira et al., 2021), but different from the other strawberry varieties, in which dark red color was mainly formed by cyanidin 3-glucoside (Dzhanfezova et al., 2020). Interestingly, the petunidin 3-*O*-rutinoside and cyanidin *O*-rutinoside were significantly down-regulated in MT at FR stage in comparison of WT (Figure 5A), revealing these anthocyanins might be more important for the coloration of WT than MT.

### Different Accumulation of PAs and Other Flavonoids

Flavan-3-ols, namely PAs and their monomers such as catechin and epicatechin etc., are the second-most abundant polyphenols after anthocyanins in strawberry fruit (Nile and Park, 2014). They are commonly considered beneficial to human health, also may act both as antifungal compounds to extend fruit shelf life, and as antioxidants to enhance fruit quality preservation (Rauf et al., 2019; Yu et al., 2020). In this study, all the DA PAs components including catechin, epicatechin and their derivatives, as well as the procyanidin A2 and B1 were significantly up-regulated in MT comparing with WT (Figure 5B). The presence of higher contents of PAs in MT (Figures 1J, 6F) probably conferred higher fruit quality, longer shelf-life and higher resistance to gray mold disease of MT fruits. Because strawberry PAs content could be used as an indicator for these important economical traits (Hébert et al., 2002). In addition, although the PAs are colorless, they can impart the color formation. The theaflavin is the main colored oxidation product of catechin, and responsible for the bright red color of black tea (Sang, 2016). Here, the theaflavin was detected in a much higher level in MT than that in WT, which was the most up-regulated one among all the DA PAs. These results indicated the PAs especially theaflavin might be involved in the coloration of MT fruits.

### The Alteration of Flavonoids in Mutant and ‘Benihoppe’ Strawberry

The changes of flavonoids during strawberry fruit development and ripening are resulted from the changes in expression of biosynthetic genes, which was mainly regulated by TFs such as MYB, bHLH etc. In this study, most of the flavonoid biosynthetic genes (such as PAL, C4H, CHI, CHS, F3H, DFR, ANS, and GST) were largely up-regulated in mutant (Figure 8), leading to the enhancement of flavonoid compounds. Notably, we found the transcription level of F3′H (FxaC_17g18710) was increased, while F3′5′H (FxaC_19g00600) expression was significantly decreased respectively at FR and PR stages in MT, leading to an alteration from delphindin to the cyanidin branch (decrease of petunidin 3-O-rutinoside and increase of cyanidin 3-O-rutinoside). In addition, the genes (FxaC_13g10950, FxaC_14g09250, FxaC_15g09560 and FxaC_16g22400) encoding LAR, being one of the key enzymes in PAs biosynthesis (Liu et al., 2016; Yu et al., 2019), were largely up-regulated in MT at FR stage (Figure 8), which could lead to the up-regulation of PAs in MT at FR stage.

Our subsequent correlation analysis revealed high correlation between flavonoid biosynthetic genes and TFs. Noticeably, the well-known TF MYB10 (FxaC_2g30690), which was suggested as the main regulator of anthocyanin biosynthesis in strawberry (Castillejo et al., 2020), showed high correlation with 40 key genes involved in flavonoids biosynthesis such as ANR, PAL, 4CL, DFR, and ANS, etc., (Supplementary Table S5). This result indicated the sufficient of correlation-based network analysis. Likewise, the high correlation of other TFs (i.e., WRKY, TCP, bZIP, and MADS etc.) and biosynthetic genes suggested their involvement in flavonoid alteration in MT fruits comparing with WT strawberry fruits (Supplementary Table S6). For the obviously enhanced PAs including procyanidin B1, (–)-epigallocatechin and epigallocatechin, the up-regulated TFs belonging to C3H, MADS, and AP2/ERF families were found highly correlated with them during the mutant fruit development and ripening in our results (Figure 9, Supplementary Table S6). The C3H proteins were proven to participate in regulation of growth, developmental processes, and environmental responses in plants, they have recently suggested to be involved in MeJA-induced flavonoid accumulation (Premathilake et al., 2020). Similarly, the MADS and ERF TFs were also reported as regulators related to anthocyanin biosynthesis (Jaakola et al., 2010; An et al., 2020), our present results provided potential evidence for their regulatory role of PAs in strawberry.

**Figure 10:**
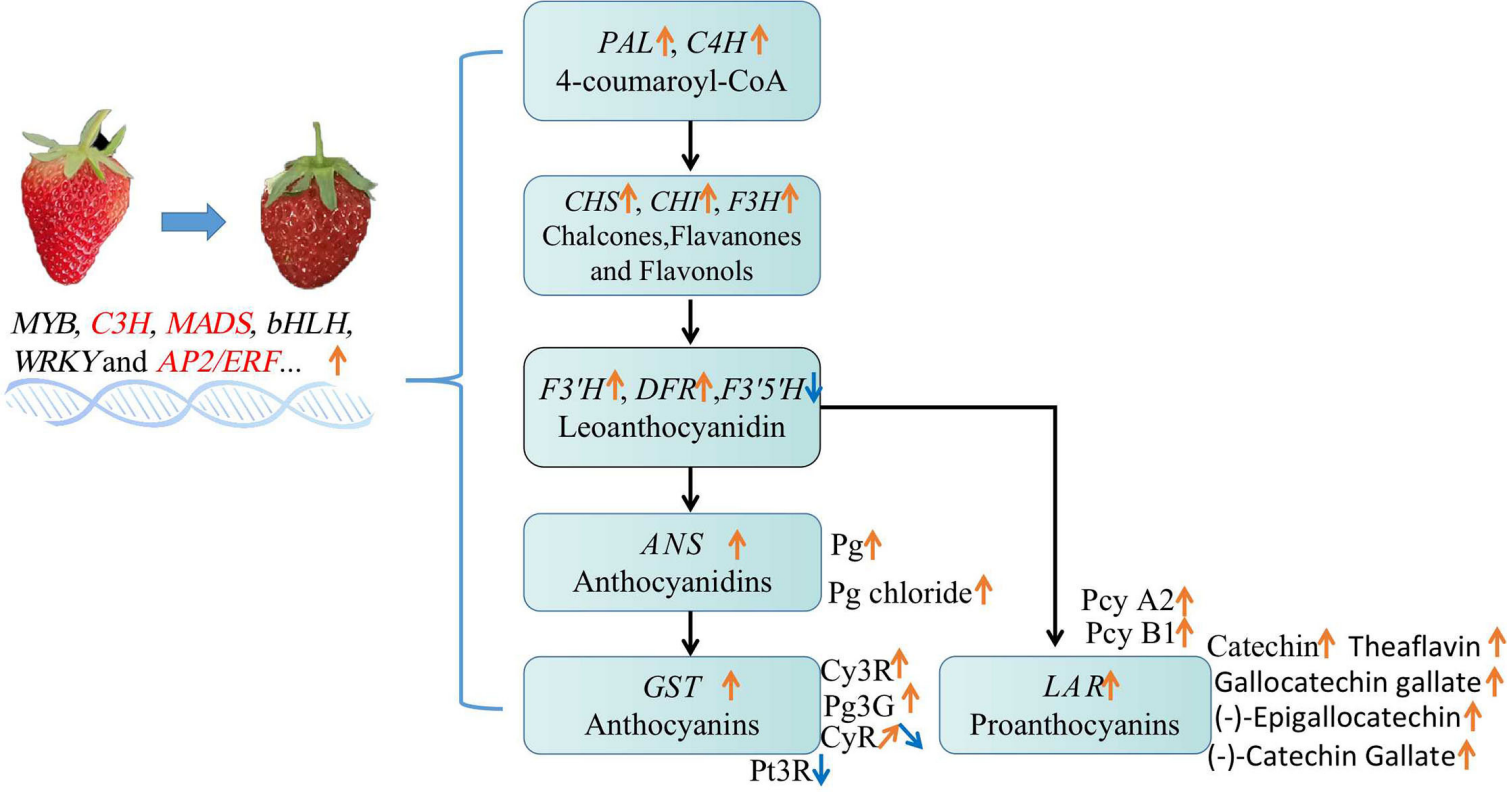
The alteration of flavonoid compounds in MT comparing with WT fruits. Changes of the gene expression and metabolites involved in flavonoid biosynthesis pathway were listed. Pg, pelargonidin; Cy3R, cyanidin 3-*O*-rutinoside; Pg3G, pelargonidin 3-glucoside; Pt3R, petunidin 3-*O*-rutinoside; CyR, cyanidin *O*-rutinoside; Pcy, procyanidin. Orange arrows represented up-regulation, blue arrows indicated down-regulation.

A summarized model showing the alteration of anthocyanins and PAs accumulation in MT comparing with WT, has been proposed and showed in Figure 10. In the mutant, various TFs were up-regulated and significantly correlated with anthocyanins and PAs during fruit development and ripening. These up-regulated TFs promoted the up-regulation of most flavonoids biosynthetic genes, and finally resulted increase of total anthocyanins and PAs. In particular, the petunidin 3-*O*-rutinoside was decreased while cyanidin 3-*O*-rutinoside and PAs were largely increased, possibly because of the reduction of *F3*′*5*′*H* but enhancement of *F3'H* and *LAR* expression (Figure 10).

## Data Availability Statement

The data presented in the study are deposited in the NCBI SRA database, accession number PRJNA838938.

## Author Contributions

YLi, HT, and YLu: conceptualization. GH and YJ: data curation. ML and YuZ: formal analysis. YLo, MY, LW, and XL: investigation. QC and YoZ: software. YLi: writing–original draft. YuZ, YW, XW, HT, and YLu: writing–editing. HT and YLu: supervision. All authors read and approved the final manuscript.

## Funding

This research was funded by the National Natural Science Foundation of China (3180817); Sweetpotato and Leguminosae Germplasm Innovation and Utilization Key Laboratory of Sichuan Province (2021CGIHL05). The funders had no role in the design of the study in the collection, analyses, or interpretation of data, in the writing of the manuscript, or in the decision to publish the results.

## Conflict of Interest

The authors declare that the research was conducted in the absence of any commercial or financial relationships that could be construed as a potential conflict of interest.

## Publisher's Note

All claims expressed in this article are solely those of the authors and do not necessarily represent those of their affiliated organizations, or those of the publisher, the editors and the reviewers. Any product that may be evaluated in this article, or claim that may be made by its manufacturer, is not guaranteed or endorsed by the publisher.

## Supplementary Material

Supplementary Figure S1 qPCR validation of selected genes MT and WT fruit.

Supplementary Table S1 Classes and peak areas of the identified flavonoids compounds.

Supplementary Table S2 Pearson correlation analysis of differentially accumulated flavonoids and total phenolic content.

Supplementary Table S3 FPKM values of flavonoid biosynthetic genes.

Supplementary Table S4 List of differentially expressed transcription factors.

Supplementary Table S5 Correlation pairs of genes and metabolites, transcription factors and genes.

Supplementary Table S6 The correlated pairs of selected up-regulated transcription factors and differentially accumulated flavonoids.

Supplementary Table S7 All primers used for qPCR validation in this study.
