# Supplementary figures and images for: Joint Transcriptomic and Metabolomic Analysis Reveals Differential Flavonoid Biosynthesis in a High-Flavonoid Strawberry Mutant
Source: Front Plant Sci. 2022 Jun 28;13:919619. doi: 10.3389/fpls.2022.919619 (PMC9274175; doi:10.3389/fpls.2022.919619)

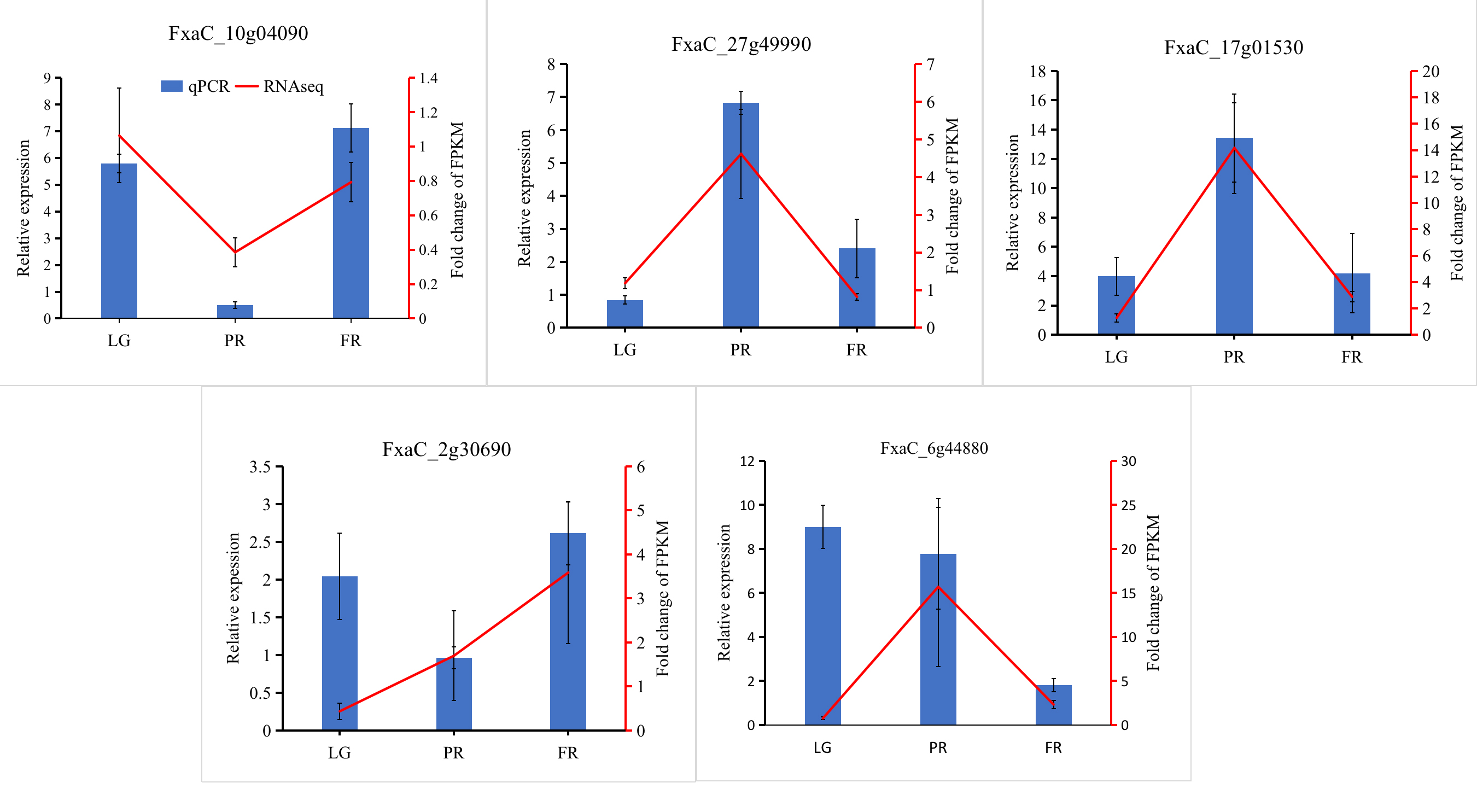

Supplement: Supplementary Figure S1 — qPCR validation of selected genes MT and WT fruit. [file Image_1.JPEG]
